# The Metric System of Measurements

**Published:** 1878-07

**Authors:** 


### Editorial.

---

It must be now evident to every careful student of science, that the irresistible tendency of the time is toward the adoption of a uniformity of standards in nomenclature, and in the measurement of weight, dimension and temperature. The action of the National Government in legalizing the metric system in this country, and the recommendation, and in some instances the actual adoption, of the system by scientific bodies in America, taken in connection with the increasing number of papers which urge the necessity of such action, and explain its methods, are significant signs of the times.

However slow may be the process by which the metric system shall be introduced into the shop and the market, the intelligent members of the medical profession are evidently determined that its standards shall be observed, at least in the records and literature of science. It is clear that the adoption of the system will become general, first among the professional men of our country. They will thus very greatly contribute to its adoption in the other fields where its necessity is so apparent.

It is better to lead than to be left behind in any movement which contemplates so excellent a result. We are impressed with the fact that the Medical Journals of the United States will feel the obligation of being the great educators of their readers in this particular; and the CHICAGO MEDICAL JOURNAL AND EXAMINER, proposes to be in the advance of a movement of the proposed character.

We have therefore to announce that, hereafter, the metric system will be exclusively adopted in these pages in the printing of prescriptions and in the record of temperature and dimensions.

We beg our readers, and especially our correspondents, to take notice of this fact. In order to render the international metric system more easy of adoption, we propose to keep standing in each number of the JOURNAL, a few simple rules for the change of ounces, scruples and drachms into their equivalents in grams and centigrams, rules for the change of Centigrade degrees into those of the Fahrenheit scale, and a space ruled in centimetres and millimetres for ready reference.

And we conclude by urging those who may, at first thought, be dissatisfied with the prospect, to remember that the sooner they familiarize themselves with the system, the sooner they will learn to read what is destined to be the international language of science the world over.

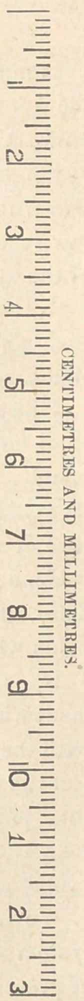

---

### THE METRIC SYSTEM IN MEDICINE.

| OLD STYLE. | | METRIC. *Gms.* |
|---|---|---|
| ♏ i or gr. i | equals | 06 |
| f ʒ i or ʒ i | " | 4 |
| f ℥ i or ℥ i | " | 32 |

The decimal *line* instead of *points* makes errors impossible.

C. C. used for Gms. causes an error of five per cent. [excess.]

A teaspoon is 5 Gms.; a tablespoon, 20 Gms.

---

SEDATIVE LOTION TO BE USED ON THE BROW IN PAINFUL AFFECTIONS OF THE CORNEA:

Extract of belladonna........... 1 gram.
Extract of hyoscyamus.......... 2 grams.
Distilled water.....................150 grams.

—(Formulary of *L'Union Médicale.*)

